# Exploring the impact of professional soccer substitute players on physical and technical performance

**DOI:** 10.1186/s13102-023-00752-x

**Published:** 2023-10-28

**Authors:** Pengyu Pan, Fangbin Li, Bo Han, Bo Yuan, Tianbiao Liu

**Affiliations:** 1 College of Physical Education and Sports, Beijing Normal University, Beijing, 100875 China; 2 China Football College, Beijing Sport University, Beijing, 100084 China

**Keywords:** Football, Soccer, Physical performance, Technical performance, Substitutions, Playing positions

## Abstract

**Background:**

Substitutions are generally used to promote the match performance of the whole team. This study aimed to analyze the performance of substitute players and explore the performance difference among substitute players, completed players, and replaced players across each position.

**Methods:**

Chinese Super Soccer League (CSL) matches in the season 2018 including 5871 individual observation from 395 professional soccer players were analyzed by establishing linear mixed models to quantify the performance difference among substitute players (SP) (*n* = 1,071), entire match players (EMP) (*n* = 3,454), and replaced players (RP) (*n* = 1,346), and then separately for each position (central defenders, fullbacks, central midfielders, wide midfielders, and attackers).

**Results:**

The results show SP display higher high intensity distance and sprint distance significantly (*p* < 0.05) relative to playing time than RP and EMP. SP in offensive positions (attackers, wide midfielders) showed significantly higher (*p* < 0.05) passing and organizing performance such as passes, ball control, short passes, and long passes than RP or EMP. The scoring performances of central midfielders of SP including goals, shots, and shots on target are significantly higher (*p* < 0.05) than RP or EMP. Central defenders of SP showed higher shot blocks and pass blocks (*p* < 0.05) while lower passing and organizing performance (*p* < 0.05).

**Conclusion:**

Depending on different playing positions, substitute players could indeed improve physical and technical performance related to scoring, passing, and defending as offensive substitute players can boost organizing performance and substitute defenders enhance defending performance. These could help coaches better understand substitute players’ influence on match performance and optimize the substitution tactic.

## Introduction

Soccer is an intermittent team sport, in which low-intensity activities dominate [1]. But the ability to perform high-intensity actions is one of the most important characteristics of professional soccer players [1–4]. Soccer players’ physical performance would decline from the first to the second half of elite soccer match play [5–7]. The reduction of players’ physical performance might be the consequence of fatigue [8–10], pacing strategy [11–13], tactical decisions from coach, contextual variables and others [5]. All the factors influence players’ performance jointly while physical fatigue seems to be an essential element evidently [5, 14, 15]. Consequently, the limited substitution numbers could change the match by coaches’ efficient substitution tactics [16]. Thus, managing the starting and substitute players is the main goal of the coach's substitutions in order to boost the team's performance and win the game[4, 17]. Soccer coaches may use substitutes in order to change tactics, replace injured or underperforming players, give playing time to inexperienced players or players coming back from injuries, or just waste time in the last few minutes [1].

Several research has investigated the physical performance of substitute soccer players, especially players’ running performance [5, 18–20]. Liu, Wang [18] also found substitute players spent more time running and less time walking or jogging than the players who were replaced and completed the entire match. In recent literature, substitute players have demonstrated higher running performance at high intensity running distance [5, 19]. Total distance and high intensity running are the variables most frequently studied, and high intensity running is one of the most representative indicators of physical performance during match-day [10]. Seminal work employing time-motion analysis find that elite substitutes who were introduced during the second half covered 25% more high intensity running distance and 63% more sprinting distance [10], and subsequent research confirm the patterns [5, 11, 21]. Additionally, in the research of England Premier League, substitute fullbacks and central midfielders sprint more than players in the same position completing the entire match, and highlights position is a key variable in soccer match performance study [5].

As crucial as the physical performance of players, technical performance is considered as a vital factor for match success and discriminant the successful team [22, 23]. However, only several pieces of literature have concentrated on the technical performance of substitute players [5, 19]. Bradley’s [5] research has studied the passing performance of substitution players but there is no evident difference. A subsequent study explored the technical performance difference between substitution and starting players in the German Bundesliga finding substitutes in midfield and attack positions display more possession, touches, and shots than replaced and entire match players [19]. Moreover, the match outcome has also been correlated with players' technical performance across all playing positions [24]. Literature suggests recent research has a tendency to pay greater attention to the technical performance of substitutes, and investigating more comprehensive technical indicators of substitutes is important [1].

Accordingly, the literature suggests fatigue, as a crucial factor, influences players’ technical performance [1]. Nonetheless, the decline in soccer players’ technical performance may be influenced by contextual variables including team strength, quality of opposition, and match location [25, 26]. Nowadays, the Chinese Soccer Super League (CSL) is growing rapidly, and performance evaluation of CSL has been progressing [27]. Consequently, quantifying physical and technical indicators of CSL substitutes in varieties of positions could provide coaches with more crucial information on substitution and explore the research of substitution further. Thus, the aims of this study were to (1) analyze the technical/tactical and physical performance of substitute players, completed players, and replaced players with considering the situational variables, (2) investigate the performance difference between substitutes versus players who replaced or completed the entire match across each playing position.

## Materials and methods

### Participants

The samples of the present study were composed of 240 matches from the Chinese Soccer Super League (CSL) in the 2018 season. The CSL season usually starts in spring and ends in winter. There are 16 teams participating in the CSL, playing a balanced home and away schedule. To avoid confounding factors, the players who played under 5 min or who were dismissed by red card were excluded from the last sample. The last sample was composed of 5,871 individual observations from 395 professional soccer players competing in CSL and were divided into three groups: entire match players (EMP) (*n* = 3,454), replaced players (RP) (*n* = 1,346), and substitute players (SP) (*n* = 1,071). To protect the privacy of players and teams, all data were anonymized in accordance with the principles of the Helsinki Declaration.

### Variables and procedures

According to previous studies [5, 19], match physical performance was examined considering the variables total distance covered, high intensity running distance, and sprints distance. The variable high intensity distance is the distance of running activities in which the players' speed is greater than 19.1 km·h^−1^ and less than 23 km·h^−1^ for at least 1 s, and sprints are the distance of running activities in which the players' speed is greater than 23 km·h^−1^ for at least 1 s [27]. All the physical performance data are tracked by the semi-automatic computerized video tracking system from the French company, Amisco®. The semi-automatic system, used 16 mm film at a frequency of 5 Hz, was first used by the French national team for the FIFA World Cup in 1998 [28]. The validity and reliability of the system have been verified in previous study [28].

For the technical performance analysis, according to previous studies [29–31], the following technical indicators divided into three groups (Table 1) were taken into consideration. All the technical data for each individual observation were collected by Champdas Soccer Big Data Company (http://www.champdas.com). A semi-automatic soccer match analysis system was developed by Champdas Soccer Big data Company. Previous research had verified the reliability and validity of the Champdas Soccer Data collection and analysis system [29]. Moreover, all the variables were used to absolute values which were converted into relative values per standard match (n·min^−1^*90 min). Additionally, each player was categorized as attackers (*n* = 1,021), fullbacks (*n* = 1,022), central midfielders (*n* = 1,484), wide midfielders (*n* = 1,346), and central defenders (*n* = 998) [5]. The classifications of team strength and quality of opposition were determined by the league rankings: (top) 1st-5th ranking, (mid) 6th-12th ranking, (bot)13th-16th ranking. Due to intra- and inter-seasonal changes in team performance, categorizing league ranking is difficult, however, a general approach was used to investigate the physical-technical performances of various team qualities [25, 26, 31, 32].

**Table 1:** Match-related performance indicators

**Performance indicators related to scoring: operational definition**	
Goals	The number of times a goal has been scored when the entire ball has crossed the goal line, between the goal posts and under the crossbar, provided the shooting team has not committed a foul
Assists	The action (such as a throw or pass) of a player who enables a teammate to score a goal
Shots	An attempt to score a goal, made with any (legal) part of the body, either on or off target
Shots on target	An attempt to score a goal, which required intervention to stop the ball going in or resulted in a goal/shot that would have gone in without diversion
**Performance indicators related to passing and organizing: operational definition**	
Passes	An intentional played ball from one player to another
Pass accuracy (%)	Successful passes as a proportion of the total passes
Ball control	Keeping the possession of the ball
Key passes	The final pass assisting a shot (without scoring)
Crosses	Balls sent into the central area of the box from a wide position of the attacking third
Breakthroughs	The number of times the ball handler successfully got rid of an opposing player after dribbling past that player
Fouled	Where a player is fouled by an opponent
Short passes	Any attempted pass less of 25 yards
Long passes	Any attempted pass of 25 yards or more
**Performance indicators related to defending: operational definition**	
Fouls	Any infringement that is penalised as foul play by a referee
Offsides	Awarded to the player deemed to be in an offside position where a free kick is awarded. If two or more players are in an offside position when the pass is played, the player considered to be most active and trying to play the ball is given offside
Tackles	The action of gaining possession from an opposition player who is in possession of the ball
Interceptions	A player intercepts a pass between oppositions and prevents the opponent receiving the ball
Clearances	A player kicks or hits the ball away from the goal of his or her own team without a precise target
Pass blocks	Number of blocked passes completed
Shot blocks	Number of blocked shots completed
Situational Variables performance-related parameters: operational definition	
Match location (ML)	The competition venue is for the participating teams to play the game, which is divided into home and away
Team strength (TS)	Strength of the participating team. TS is evaluated using the ranking of the team and divided into top, middle, and bottom
Quality of opposition (QO)	Strength of the opponent team. QO is evaluated using the ranking of the team and divided into top, middle, and bottom

### Statistical analyses

All data was structured in Excel and imported into R (ver.4.1.1) (Vienna, Austria) for pre-processing. The descriptive results are reported as mean ± standard deviation (SD) for each variable (Table 2). Linear mixed models were built using the R packages lmerTest [33] to analyze the performance differences between substitute, replaced, and entire match players, and the performance indicators (PI) were designed as dependent variables. The types of player (ToP) was designed as independent variables while match location (ML), team strength (TS), and quality of opposition (QO) were designed as covariates, and players were deemed as random effect. The linear mixed models were established:

**Table 2:** Descriptive statistics of match performance indicators among SP, RP, and EMP (mean ± SD)

		SP	RP	EMP
		(*n* = 1,071)	(*n* = 1,346)	(*n* = 3,454)
Physical performance indicators	TD (m)	106.275 ± 13.082	113.658 ± 11.773	103.126 ± 9.488
	HID (m)	7.022 ± 3.375	6.108 ± 2.615	4.863 ± 2.156
	SPD (m)	3.162 ± 2.194	2.662 ± 1.591	2.179 ± 1.277
Technical performance indicators	Goals	0.187 ± 1.126	0.161 ± 0.485	0.150 ± 0.416
	Assists	0.077 ± 0.583	0.120 ± 0.385	0.103 ± 0.338
	Shots	1.529 ± 3.284	1.195 ± 1.620	1.251 ± 1.649
	SoT	0.551 ± 1.870	0.424 ± 0.867	0.455 ± 0.846
	Passes	34.279 ± 24.638	33.756 ± 14.694	36.271 ± 15.634
	PA (%)	71.048 ± 26.643	73.210 ± 12.872	75.252 ± 11.331
	Ball controls	126.437 ± 88.920	120.233 ± 51.072	137.019 ± 58.477
	Key passes	0.975 ± 2.369	0.733 ± 1.102	0.829 ± 1.159
	Crosses	2.120 ± 3.822	1.675 ± 2.334	1.613 ± 2.417
	Breakthroughs	1.430 ± 3.215	1.363 ± 1.895	1.210 ± 1.867
	Fouled	1.542 ± 3.139	1.366 ± 1.541	1.276 ± 1.412
	Short passes	31.676 ± 22.821	31.068 ± 13.592	32.723 ± 14.654
	Long passes	2.410 ± 3.740	2.642 ± 2.673	3.631 ± 2.741
	Fouls	2.302 ± 3.591	1.610 ± 1.613	1.347 ± 1.223
	Offsides	0.267 ± 1.371	0.203 ± 0.624	0.179 ± 0.531
	Tackles	1.781 ± 3.365	1.469 ± 1.603	1.536 ± 1.445
	Interceptions	0.937 ± 2.326	0.961 ± 1.364	1.125 ± 1.310
	Clearances	1.222 ± 3.277	1.074 ± 1.774	2.257 ± 2.514
	Pass blocks	0.270 ± 1.427	0.184 ± 0.551	0.291 ± 0.588
	Shot blocks	1.075 ± 2.635	1.033 ± 1.308	0.917 ± 1.073

*Abbreviations*: *SP* Substitute player, *RP* Replaced player, *EMP* Entire match player, *TD* Total distance, H*ID* High intensity distance, *SPD* Sprint distance, *SoT* Shots on target, *PA* Pass accuracy

1 $$PI={\beta }_{0}+{\beta }_{1}\cdot {ToP}_{ijmn}+{\beta }_{2}\cdot {ML}_{j}+{\beta }_{3}\cdot {TS}_{m}+{\beta }_{4}\cdot {QO}_{n}+{\lambda }_{mn}+{\mu }_{j}+{\varepsilon }_{ijmn}$$

These analyses were run on the entire set of data first, and then independently on each playing position such as attackers (*n* = 1,021), fullbacks (*n* = 1,022), central midfielders (*n* = 1,484), wide midfielders (*n* = 1,346), and central defenders (*n* = 998). In the linear mixed models, categorical variables must be selected one category as the baseline to examine the effects on models. For the independent variable ToP, the SP is selected as the baseline to quantify the difference between SP versus RP and EMP. Each model's homogeneity and normal distribution assumptions were confirmed with no particular issues. The significance value was set at *p* < 0.05 for all analyses. To better demonstrates the technical differences among SP, RP, and EMP, the present study designed a statistic radar graph showing the model results.

## Results

Table 3 displays the running performance results of linear mixed models. Totally, SP showed higher high intensity running distance (*p* < 0.001), and sprint distance (*p* < 0.001) than RP and EMP while showing lower total distance (*p* < 0.001) than the counterparts. In the playing positions of central defenders, central midfielders, wide midfielders, and fullbacks, SP showed significantly higher high intensity distance (*p* < 0.05) and sprint distance (*p* < 0.05) than RP and EMP in each same position. However, in all playing positions, SP showed a lower total distance (*p* < 0.05) than RP or EMP.

**Table 3:** The physical performance results of liner mixed models

	Independent of Position						Central Defenders					
	SP	RP	SE	EMP	SE	F test(*p*)	SP	RP	SE	EMP	SE	F test(*p*)
	(*n* = 1,071)	(*n* = 1,346)		(*n* = 3,454)			(*n* = 62)	(*n* = 80)		(*n* = 856)		
TD (m)	baseline	771.833***	34.22	217.924***	61.56	< 0.001	baseline	684.700***	85.53	265.210**	96.38	< 0.001
HID (m)		-69.407***	8.175	-123.419***	8.175	< 0.001		-55.178*	15.709	-100.462***	18.127	< 0.001
SPD (m)		-39.935***	5.029	-64.129***	5.029	< 0.001		-26.504*	9.652	-43.211***	11.138	< 0.001
	Fullbacks						Central Midfielders					
	SP	RP	SE	EMP	SE	F test(p)	SP	RP	SE	EMP	SE	F test(*p*)
	(*n* = 54)	(*n* = 198)		(*n* = 770)			(*n* = 331)	(*n* = 333)		(*n* = 820)		
TD (m)	baseline	479.566***	113.32	100.833	95.33	< 0.001	baseline	869.110***	73.31	292.94***	58.29	< 0.001
HID (m)		-44.548	27.23	-105.097***	23.91	< 0.001		-141.607***	18.02	-160.31***	14.33	< 0.001
SPD (m)		-53.365***	17.15	-80.360***	14.43	< 0.001		-80.630***	10.294	-81.408***	8.185	< 0.001
	Wide Midfielders						Attackers					
	SP	RP	SE	EMP	SE	F test(p)	SP	RP	SE	EMP	SE	F test(*p*)
	(*n* = 456)	(*n* = 456)		(*n* = 434)			(*n* = 168)	(*n* = 279)		(*n* = 574)		
TD (m)	baseline	885.807***	68.81	326.541***	67.24	< 0.001	baseline	705.717***	99.22	254.842***	78.74	< 0.001
HID (m)		-50.492**	18.02	-102.319***	17.61	< 0.001		-37.732	22.95	-108.663***	18.21	< 0.001
SPD (m)		-31.935**	11.99	-53.816***	11.71	< 0.001		-9.932	15.405	-56.085***	12.226	< 0.001

*Abbreviations*: *SP* Substitute player, *RP* Replaced player, *EMP* Entire match player, *TD* Total distance, *HID* High intensity distance, *SPD* Sprint distance, *SE* Std. Error SP is selected as the baseline, the results demonstrate the differences compared to the baseline. Significance level: ***p < 0.001, **p < 0.01, *p < 0.05

**Fig. 1:**
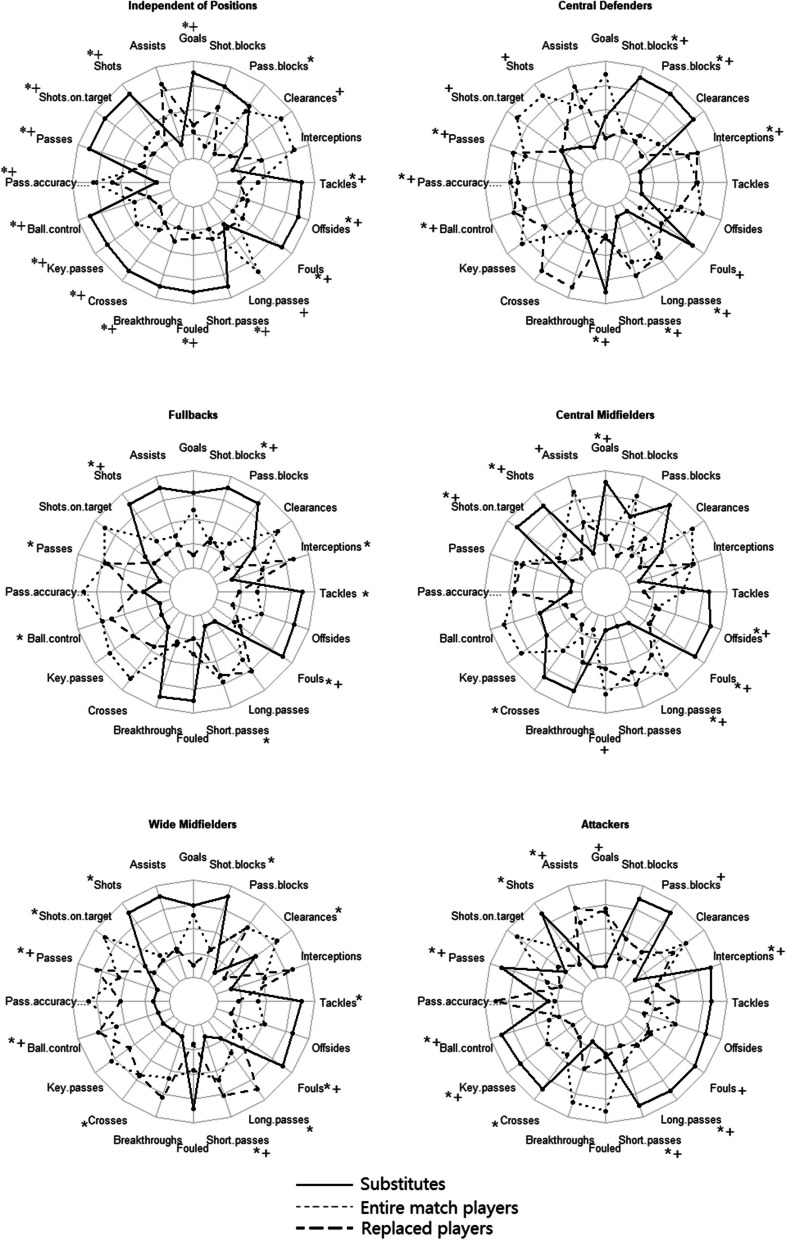
The technical performance results of linear mixed models. * Significant difference between substitutes and replaced players (*p* < 0.05). + Significant difference between substitutes and players completed the entire match (*p* < 0.05)

The technical results of models are demonstrated on the Table 4, and to better understand the technical performance difference among SP, RP, and EMP, a radar graph is designed (Fig. 1). Independent of playing positions (Fig. 1(A)), SP showed higher performance related to scoring including goals (*p* < 0.05), shots (*p* < 0.05), and shots on target (*p* < 0.05) than RP and EMP. Substitutes’ performance related to passing and organizing is significantly higher than RP and EMP excluding pass accuracy and long passes. Regarding to the defending performance, SP display higher fouls (*p* < 0.05), tackles (*p* < 0.05), offside (*p* < 0.05), and pass blocks (*p* < 0.05) than RP or EMP. Moreover, with considering to playing positions, substitute central midfielders (Fig. 1(C)) and wide midfielders (Fig. 1(E)) showed higher scoring performance such as shots (*p* < 0.05) and shots on target (*p* < 0.05) than the counterparts in the same playing positions. Although substitute attackers (Fig. 1(F)) have higher shots, SP of attackers show lower goals (*p* < 0.05) and assists (*p* < 0.05) than attackers of RP or EMP. SP in offensive positions such as attackers (Fig. 1(F)) and wide midfielders (Fig. 1(E)) show higher passing and organizing performance including passes (*p* < 0.05), ball controls (*p* < 0.05), key passes (*p* < 0.05), crosses (*p* < 0.05), short passes (*p* < 0.05), and long passes (*p* < 0.05) than RP or EMP in the same positions. SP of central defenders (Fig. 1(B)) and fullbacks (Fig. 1(D)) display lower passing and organizing performance while have higher performance of defending such as pass blocks (*p* < 0.05), tackles (*p* < 0.05), and shot blocks (*p* < 0.05) than RP or EMP in the same positions. Moreover, SP of attackers (Fig. 1(F)) also show higher defending performance including interceptions (*p* < 0.05) and pass blocks (*p* < 0.05) than the counterparts in the same positions.

**Table 4:** The physical performance results of liner mixed models

	Independent of Position			Central Defenders			Fullbacks		
	SP	RP	EMP	SP	RP	EMP	SP	RP	EMP
	(*n* = 1,071)	(*n* = 1,346)	(*n* = 3,454)	(*n* = 62)	(*n* = 80)	(*n* = 856)	(*n* = 54)	(*n* = 198)	(*n* = 770)
Goals	Baseline	-0.037	-0.026	Baseline	0.027	-0.017	Baseline	-0.007	-0.026
Assists		0.026	0.043**		0.023	0.03		-0.048	-0.056
Shots		-0.279***	-0.334***		0.263**	0.074		-0.229*	-0.309**
SoT		-0.096*	-0.127**		0.087*	-0.007		0.064	-0.014
Passes		1.992**	-0.523		12.149***	15.268***		6.725**	6.323**
PA (%)		4.203***	2.162***		13.161***	11.897***		3.941*	0.561
Ball controls		10.582***	-6.204*		38.004***	43.465		23.823***	20.127**
Key passes		-0.146**	-0.242***		0.014	-0.019		0.164	0.092
Crosses		-0.507***	-0.445***		0.029	0.109		0.325	0.121
Breakthroughs		-0.22**	-0.067		0.085	0.033		-0.574**	-0.539*
Fouled		-0.266***	-0.176		-0.593***	-0.677***		-0.507*	-0.676**
Short passes		1.047	-0.609		9.803***	12.652***		6.318***	5.582**
Long passes		1.221***	0.232		2.459***	2.505***		0.544	0.71
Fouls		-0.955***	-0.692***		-0.552**	-0.279		-0.93***	-0.82***
Offsides		-0.088**	-0.064*		0.042	0.025		-0.037	-0.069
Tackles		-0.244***	-0.312***		0.139	0.185		-0.496	-0.686*
Interceptions		0.189***	0.025		0.538**	0.636*		0.21	0.429
Clearances		1.034***	-0.149		-0.144	-0.235		0.258	-0.311
Pass blocks		0.021	-0.086*		-0.676***	-0.793***		-0.073	-0.091
Shot blocks		-0.157**	-0.042		-1.038***	-0.956***		-0.575**	-0.535*
	Central Midfielders			Wide Midfielders			Attackers		
	SP (*n* = 331)	RP (*n* = 333)	EMP (*n* = 820)	SP (*n* = 456)	RP (*n* = 456)	EMP (*n* = 434)	SP (*n* = 168)	RP (*n* = 279)	EMP (*n* = 574)
Goals	Baseline	-0.079	-0.129	Baseline	0.015	-0.041	Baseline	0.179*	0.173*
Assists		0.099***	0.04		0.069	0.038		0.071	0.109*
Shots		0.011	-0.507**		0.208	-0.318*		0.257	-0.232
SoT		-0.111	-0.306**		0.125	-0.147		0.287	0.139
Passes		7.508***	3.865*		0.328	-3.438**		-2.8*	-6.841***
PA(%)		3.9625***	3.2631**		3.107**	1.869		1.881	1.725
Ball controls		38.357***	12.552*		7.424	-24.021***		-6.603	-26.628***
Key passes		0.255**	-0.1		0.371***	-0.124		-0.343*	-0.839***
Crosses		0.378*	0.037		0.518*	-0.513*		-1.05***	-1.411***
Breakthroughs		0.267*	-0.038		0.543**	-0.043		0.515*	0.162
Fouled		0.383***	0.209		-0.12	-0.327		0.333	0.042
Short passes		6.657***	3.451*		-0.128	-2.841**		-2.441*	-6.276***
Long passes		1.559***	0.982***		0.523**	-0.627***		-0.355*	-0.63***
Fouls		-1.043***	-0.81***		-0.873***	-0.514*		-0.84***	-0.818***
Offsides		-0.168***	-0.198***		0.016	-0.013		0.017	-0.04
Tackles		0.091	-0.029		-0.238	-0.462**		-0.549***	-0.428**
Interceptions		0.23*	0.207		-0.177	-0.206		-0.321***	-0.294***
Clearances		0.261*	-0.179		-0.244	-0.43***		0.059	-0.028
Pass blocks		-0.054	-0.022		-0.033	-0.054		-0.102*	-0.082
Shot blocks		0.041	-0.083		-0.004	0.214		-0.157	-0.069

*Abbreviations*: *SP* Substitute player, *RP* Replaced player, *EMP* Entire match player, *SoT* Shots on target, *PA* Pass accuracy SP is selected as the baseline, the results demonstrate the differences compared to the baseline. Significance level: ***p < 0.001, **p < 0.01, *p < 0.05

Table 5 demonstrates the impact of situational variables, which are designed as covariates, on match performance from models. Match location have no significant influence on running performance while influencing most of technical indicators except for offside, clearances, and shot blocks. Team strength influences all physical performance indicators and mainly influences performance indicators related to scoring, passing, and organizing. Quality of opposition has significant impact on high intensity running distance and sprint distance which related to physical performance. Moreover, quality of opposition has the similar impact on technical performance with team strength such as goals, assists, shots on target, passes, pass accuracy, ball controls, short passes, and so on.

**Table 5:** The results of situational variables of liner mixed models

		TS					QO					ML		
		bot	mid	se	top	se	bot	mid	se	top	se	away	home	se
Physical performance	TD (m)	BL	61.558	91.338	203.575*	99.501	BL	-6.502	23.927	30.187	30.187	BL	24.663	18.863
	HID (m)		2.060	21.399	75.671**	23.315		12.89*	5.793	17.638**	6.174		7.067	4.567
	SPD (m)		7.565	12.076	40.292**	13.162		7.874*	3.787	9.397*	4.036		6.371	2.985
Performance related to scoring	Goals	BL	0.005	0.032	0.096**	0.035	BL	0.001	0.020	-0.048*	0.021	BL	0.051**	0.016
	Assists		0.007	0.017	0.085***	0.018		-0.027*	0.013	-0.056***	0.014		0.040***	0.010
	Shots		-0.144	0.157	0.280	0.170		0.059	0.057	-0.105	0.061		0.244***	0.045
	Shots on target		-0.020	0.072	0.165*	0.078		0.013	0.033	-0.103*	0.035		0.103***	0.026
Performance related to passing and organizing	Passes	BL	-0.246	1.323	6.978***	1.434	BL	1.573***	0.470	-0.599	0.501	BL	2.772***	0.372
	Pass accuracy (%)		3.714***	1.073	5.343***	1.165		1.96***	0.461	0.681	0.491		1.159**	0.365
	Ball control		-3.887	5.072	17.574**	5.493		4.074*	1.678	-2.773	1.792		10.579***	1.330
	Key passes		-0.059	0.088	0.095	0.095		-0.006	0.047	-0.062	0.047		0.171***	0.035
	Crosses		-0.668**	0.225	0.121	0.243		0.018	0.069	0.043	0.073		0.273***	0.054
	Breakthroughs		0.056	0.174	0.213	0.189		0.070	0.060	0.037	0.064		0.130**	0.047
	Fouled		0.036	0.141	0.009	0.153		-0.039	0.055	-0.031	0.059		0.098*	0.044
	Short passes		0.213	1.205	6.894***	1.307		1.572***	0.441	-0.401	0.471		2.504***	0.350
	Long passes		-0.44	0.230	-0.243	0.249		-0.040	0.081	-0.217*	0.087		0.172**	0.064
Performance related to defending	Fouls	BL	-0.071	0.104	0.091	0.112	BL	0.116	0.062	0.006	0.066	BL	-0.079	0.049
	Offsides		0.023	0.042	0.050	0.046		-0.083***	0.024	-0.077**	0.026		0.001	0.019
	Tackles		0.014	0.105	0.255*	0.114		0.097	0.061	0.163*	0.066		-0.184***	0.049
	Interceptions		-0.054	0.084	-0.057	0.092		-0.016	0.049	0.042	0.052		-0.020	0.039
	Clearances		-2.850	0.208	-0.275	0.225		-0.200	0.068	-0.096	0.073		-0.225***	0.054
	Pass blocks		-0.007	0.040	-0.023	0.043		0.039	0.026	0.035	0.027		-0.056**	0.020
	Shot blocks		-0.184*	0.088	-0.153	0.096		-0.063	0.048	-0.047	0.051		-0.072	0.038

*Abbreviations*: *TD* Total distance, *HID* High intensity distance, *SPD* Sprint distance, *SE* Std. Error, *BL* Baseline, *TS* Team strength, *QO* Quality of opposition

Significance level: ***p < 0.001, **p < 0.01, *p < 0.05

## Discussion

The present study aimed to (1) quantify the physical and technical profile of substitutes, replaced players, and players who completed the entire match considering situational variables; (2) analyze the physical and technical performance difference of substitutes versus players who replaced or completed the entire match across each playing position. Previous studies have mainly investigated the physical performance of substitutes in soccer [5, 18–21], but only a few have explored the technical performance of substitutions [5, 19]. The current study analyzed more ample and detailed technical indicators to investigate the impact of substitutes on technical performance. The results of the current study have demonstrated that substitute players perform better in physical performance (Table 3), especially high intensity running distance and sprint distance than players who were replaced or completed the entire match. The findings have shown the similarity with previous studies as the better physical performance of substitute players [5, 19]. Substitutions are generally used to reduce the influence of fatigue and maintain the high level running performance of the whole team [34]. High intensity running distance has seemed as an especially essential and useful indicator of physical performance in soccer [35], and the findings of the present study showed the significant difference on high intensity running distance between substitutes and players who are replaced or complete the entire match. However, substitute players show lower performance on total distance than replaced players. Accordingly, it is the major factor that the length of time playing on the pitch influence match intensity [36]. Replaced players mainly play on the pitch in the first half and would be replaced in the second half [5]. Thus, during the playing time of replaced players, the match intensity may be higher than substitutes playing on the pitch resulting in substitutes showing lower total distance. Depending on playing positions, substitutes of all positions show higher high intensity distance and sprint distance than replaced players or players complete the entire match. The finding is similar to the results independent of positions. However, wide midfielder substitutes display lower total distance than replaced players and players completed the entire match. Coaches would introduce more defensive players to strengthen the defense when the team is winning [4]. Modric, Versic [37] found running total distance of wide midfielders decreased and sprint distance increased in the defending phases which are in agreements with the findings of the present study.

Concentrating on technical performance, technical indicators in a broader range have been analyzed, and quantified the performance related to scoring, passing, and defending (Table 1). Despite the importance of technical performance, only few pieces of literature have paid attention to substitute players’ technical activities [1, 5, 19]. Bradley, Peñas [5] first analyzed the technical activities of substitute players while finding no significant difference in passing activities between replaced players and players who completed the entire match. The results of the present study show substitutes mainly perform better on shooting activities, and defending activities while showing worse passing activities including passes, pass accuracy, and long passes than players those who replaced or completed the entire match (Fig. 1). Existing literature has reported passing activities such as short passes, successful passes decrease from the first to the second half of soccer matches which might be influenced by fatigue [38]. Under the theory, it is a good strategy for the coach to introduce substitutions on the pitch to counteract the technical performance decline of the team. Furthermore, match status also influences players’ performance, as substitute players might attempt more risky passes and crosses due to the match status when they were introduced onto the pitch [39]. Additionally, research on substitution introduction patterns finds that coaches would introduce more players of offensive positions in the second half of the match [5]. Thus, substitute players would try more risky and offensive activities when coaches aim to change the score line, resulting substitute players act lower pass accuracy but more offensive activities including shots, shots on target, key passes, and breakthroughs.

One of the most compelling findings in time-motion analysis research is the significant differences between all playing positions in physical performance [6–8, 40–42] and technical performance [5, 24, 43, 44] of elite soccer players. Hence, it is crucial to discuss the performance differences between players who are substituted, replaced, and completed the entire match for each playing position. According to this study, substitute central defenders have shown lower passing and organizing activities whereas performing more defending actions. In order to win the match, coaches generally send the defenders onto the pitch when their team is ahead [16, 17]. Thus, the tactical aim of substitute central defenders and fullbacks is to defend the opponents’ attackers and protect the attacking third zone. Under the tactical purpose, substitute defenders (central defenders and fullbacks) would perform lower passing actions and higher defending actions compared with players who replaced or completed the entire match. Additionally, for strengthening the defending level, offensive players may also have been introduced onto the pitch as defending roles, in order to hold the scoring line or waste time during the final stages of the match [1]. Those could be the reasons why offensive substitute players such as attackers and wide midfielders have displayed more tackles, clearances, and pass blocks than players who were replaced or completed the entire match.

On the other hand, the present study has found substitute attackers and wide midfielders show better passing and organizing actions such as passes, ball controls, crosses, short passes, and long passes than players who were replaced or completed the entire match. Players of offensive playing positions would typically be introduced to the pitch when their team is losing [1, 16, 17]. In general, replaced players are more likely to be deemed underperforming when the team is losing [17], and coaches propose offensive substitute players to create more scoring opportunities and improve the performance of the whole team [5, 39]. The higher passing and organizing performance of offensive substitutes than players who were replaced or completed the match is deemed to be crucial factors for the match success [5, 24, 39, 45]. Moreover, substitute central midfielders have displayed more scoring actions in this research. Research on French elite soccer has found the increased number of goals from substitutes was a factor in discriminating successful teams [46]. Hence, substitute central midfielders may be introduced to the pitch as another attacker to score a goal when their team is losing. Those could be reasons why substitutes central midfielders perform lower passing performance than players replaced and completed the entire match. Additionally, one very interesting result of the present study is that substitute fullbacks perform more shots, which is more likely to be the offensive playing style. Although coaches introduce some players of defensive positions like fullbacks, the introduced players may play in the offensive playing position trying to create scoring opportunities [19].

Overall, substitute players indeed can improve the physical and technical performance of the team. The present study has confirmed previous findings, as substitutes are introduced to change the score line, or reduce the influence of fatigue [1, 4, 5, 16, 17, 19, 39, 47]. With considering on the situational variables, the current study analyzed a broader range of technical variables and quantified the technical performance of substitutes across different playing positions. Moreover, match location influences most of the technical performance indicators showing higher scoring and passing performance while lower defending performance. The findings have verified the home advantage and confirmed previous research [26, 31].

However, some limitations should be studied further in later research. Literature on sports psychology found that substitutes might perform worse when they are introduced as a starter in the match because of the psychological burden [48]. It is important to study the performance difference of substitutes between the substitution situations and the starter situation. Moreover, the timing of substitutes introduced to the pitch also influences the performance, as the match status determines the tactical purpose of substitutions [5, 16, 17]. The present study only analyzed the match data for one year and the substitution option has been changed in 2020. To better investigate the influence of substitute players on match performance, it is important for future study to analyze match data in a more range of seasons.

## Conclusions

The present research has demonstrated that substitute soccer players could improve the performance of the team, not only the physical performance but also technical performance based on different playing positions. In general, substitutions are the important way for coaches to directly affect the soccer matches. This study can help professionals to better understand the effects of substitutions on professional soccer leagues, especially in the CSL. By the way, the findings, based on the three-substitute option, might not provide enough experiences for current soccer matches, using five-substitute option whereas it is a useful approach to investigate the substitution option.

## Data Availability

The data that support the findings of this study are available from Champdas Football Big Data Company (http://www.champdas.com) and Amisco®, but restrictions apply to the availability of these data, which were used under license for the current study, and so are not publicly available. Data are however available from the authors upon reasonable request and with permission of Champdas Football Big Data Company and Luneng football club. If someone wants to study further with the data, please contact the corresponding author.

## Abbreviations

CSL: The Chinese football Super League
PI: Performance indicator
SP: Substitute player
RP: Replaced player
EMP: Entire match player
ToP: The type of players
ML: Match location
TS: Team strength
QO: Quality of opposition

## References

[1] Hills S, Barwood M, Radcliffe J (2018). Profiling the Responses of Soccer Substitutes: A Review of Current Literature. Sports Med.

[2] Bangsbo J, Iaia F, Krustrup P (2007). Metabolic Response and Fatigue in Soccer. Int J Sports Physiol Perform.

[3] Bangsbo J, Mohr M, Krustrup P (2006). Physical and metabolic demands of training and match-play in the elite football player. Nutri Football.

[4] Del Corral J, Barros C, Prieto-Rodriguez J (2008). The Determinants of Soccer Player Substitutions: A Survival Analysis of the Spanish Soccer League. J Sports Econ.

[5] Bradley P, Peñas C, Rey E (2014). Evaluation of the Match Performances of Substitution Players in Elite Soccer. Int J Sports Physiol Perform.

[6] Bradley P, Sheldon W, Wooster B (2009). High-intensity running in English FA Premier League Soccer Matches. J Sports Sci.

[7] Di Salvo V, Gregson W, Atkinson G (2009). Analysis of High Intensity Activity in Premier League Soccer. Int J Sports Med.

[8] Bradley P, Di Mascio M, Peart D (2009). High-Intensity Activity Profiles of Elite Soccer Players at Different Performance Levels. J Strength Cond Res.

[9] Krustrup P, Mohr M, Steensberg A (2006). Muscle and Blood Metabolites during a Soccer Game. Med Sci Sports Exerc.

[10] Mohr M, Krustrup P, Bangsbo J (2003). Match performance of high-standard soccer players with special reference to development of fatigue. J Sports Sci.

[11] Bradley P (2013). Match running performance fluctuations in elite soccer: Indicative of fatigue, pacing or situational influences?. J Sports Sci.

[12] Drust B, Atkinson G, Reilly T (2007). Future Perspectives in the Evaluation of the Physiological Demands of Soccer. Sports Med.

[13] Edwards A (2009). Dehydration Cause of Fatigue or Sign of Pacing in Elite Soccer?. Sports Med.

[14] Krustrup P, Zebis M, Jensen J (2010). Game-Induced Fatigue Patterns in Elite Female Soccer. J Strength Cond.

[15] Mohr M, Krustrup P, Nybo L (2004). Muscle temperature and sprint performance during soccer matches - Beneficial effect of re-warm-up at half-time. Scand J Med Sci Sports.

[16] Rey E, Lago Ballesteros J, Padrón CA (2015). Timing and tactical analysis of player substitutions in the UEFA Champions League. Int J Perform Anal Sport.

[17] Myers B (2012). A Proposed Decision Rule for the Timing of Soccer Substitutions. J Quant Anal Sports.

[18] Liu H, Wang L, Huang G (2019). Activity profiles of full-match and substitution players in the 2018 FIFA World Cup. Eur J Sport Sci.

[19] Lorenzo-Martínez M, Padrón Cabo A, Rey E, et al. Analysis of Physical and Technical Performance of Substitute Players in Professional Soccer. Res Quart Exerc Sport. 2020;92:599–606. 10.1080/02701367.2020.1755414. 10.1080/02701367.2020.1755414 32603634

[20] Padrón Cabo A, Rey E, Vidal B (2018). Work-rate Analysis of Substitute Players in Professional Soccer: Analysis of Seasonal Variations. J Human Kinetics..

[21] Carling C, Espié V, Gall F (2009). Work-rate of substitutes in elite soccer: A preliminary study. J Sci Med Sport.

[22] Castellano J, Casamichana D, Peñas C (2012). The Use of Match Statistics that Discriminate Between Successful and Unsuccessful Soccer Teams. J Hum Kinet.

[23] Peñas C, Lago Ballesteros J, Rey E (2011). Section III – Sport, Physical Education & Recreation Differences in performance indicators between winning and losing teams in the UEFA Champions League. J Hum Kinet.

[24] Konefał M, Chmura P, Zając T (2019). Evolution of technical activity in various playing positions, in relation to match outcomes in professional soccer. Biol Sport.

[25] Almeida C, Ferreira A, Volossovitch A (2014). Effects of Match Location, Match Status and Quality of Opposition on Regaining Possession in UEFA Champions League. J Hum Kinet.

[26] Peñas C (2009). The influence of match location, quality of opposition, and match status on possession strategies in professional association football. J Sports Sci.

[27] Zhou C, Ruano M, Lorenzo Calvo A (2020). The evolution of physical and technical performance parameters in the Chinese Soccer Super League. Biol Sport..

[28] Castellano J, Alvarez-Pastor D, Bradley P (2014). Evaluation of Research Using Computerised Tracking Systems (Amisco® and Prozone®) to Analyse Physical Performance in Elite Soccer: A Systematic Review. Sports medicine (Auckland, NZ)..

[29] Gong B, Cui Y, Gai Y (2019). The Validity and Reliability of Live Football Match Statistics From Champdas Master Match Analysis System. Front Psychol.

[30] Han B, Yang L, Pan P, et al. The influence of removing home advantage on the Chinese Football Super League. BMC Sports Sci Med Rehabil. 2022;14(1):208. 10.1186/s13102-022-00604-0. 2022/12/11. 10.1186/s13102-022-00604-0 PMC9733305 36494746

[31] Liu T, Garcia de Alcaraz A, Zhang L (2019). Exploring home advantage and quality of opposition interactions in the Chinese Football Super League. Int J Perform Anal Sport.

[32] Ju W, Hawkins R, Doran D (2022). Tier-specific contextualised high-intensity running profiles in the English Premier League: more on-ball movement at the top. Biol Sport.

[33] Kuznetsova A, Brockhoff P, Christensen R (2007). LmerTest: Tests in linear mixed effects models. J Stat  Softw.

[34] Reilly T, Drust B, Clarke N (2008). Muscle fatigue during football match-play. Sports Med..

[35] Bangsbo JL, Nørregaard L, Thorsø F (1991). Activity profile of competition soccer. Can J Sport Sci.

[36] Waldron M and Highton J. Fatigue and Pacing in High-Intensity Intermittent Team Sport: An Update. Sports medicine (Auckland, NZ) 2014; 44. 10.1007/s40279-014-0230-6. 10.1007/s40279-014-0230-6 25047854

[37] Modric T, Versic S, Drid P (2021). Analysis of Running Performance in the Offensive and Defensive Phases of the Game: Is It Associated with the Team Achievement in the UEFA Champions League?. Appl Sci.

[38] Rampinini E, Impellizzeri F, Castagna C (2007). Technical performance during soccer matches of the Italian Serie A league. J Sci Med Sport.

[39] Ruano M, Peñas C, Owen A (2016). The influence of substitutions on elite soccer teams' performance. Int J Perform Anal Sport.

[40] Bradley P, Carling C, Archer D (2011). The effect of playing formation on high-intensity running and technical profiles in English FA Premier League soccer matches. J Sports Sci.

[41] Carling C (2010). Analysis of physical activity profiles when running with the ball in a professional soccer team. J Sports Sci.

[42] Di Salvo V, Baron R, Tschan H (2007). Performance Characteristics According to Playing Position in Elite Soccer. Int J Sports Med.

[43] A D, Wong DP, Moalla W, et al. Physical and technical activity of soccer players in the French First League – with special reference to their playing position. Int Sport Med J 2010; 11: 278–290.

[44] Konefał M, Chmura P, Chmura J, et al. Evolution of technical activities regarding match outcomes for various playing positions in the Bundesliga. 2018.

[45] Bradley P, Peñas C, Rey E (2013). The effect of high and low percentage ball possession on physical and technical profiles in English FA Premier League soccer matches. J Sports Sci.

[46] Carling C, Gall F, McCall A (2014). Squad management, injury and match performance in a professional soccer team over a championship-winning season. Eur J Sport Sci.

[47] Hills S, Barrett S, Feltbower R (2019). A match-day analysis of the movement profiles of substitutes from a professional soccer club before and after pitch-entry. PLoS ONE.

[48] Dancy B. An Exploration of Substitutes' Experiences in Football. 2007.

